# Successful conversion surgery of distal pancreatectomy with celiac axis resection (DP-CAR) with double arterial reconstruction using saphenous vein grafting for locally advanced pancreatic cancer: a case report

**DOI:** 10.1186/s40792-020-01082-7

**Published:** 2020-12-01

**Authors:** Yoshiki Murase, Daisuke Ban, Aya Maekawa, Shuichi Watanabe, Yoshiya Ishikawa, Keiichi Akahoshi, Kosuke Ogawa, Hiroaki Ono, Atsushi Kudo, Toshifumi Kudo, Shinji Tanaka, Minoru Tanabe

**Affiliations:** 1 Department of Hepatobiliary and Pancreatic Surgery, Graduate School of Medicine, Tokyo Medical and Dental University, 1-5-45 Yushima, Bunkyo-ku, Tokyo, 113-8519 Japan; 2 Division of Vascular and Endovascular Surgery, Department of Surgery, Tokyo Medical and Dental University, 1-5-45 Yushima, Bunkyo-ku, Tokyo, 113-8519 Japan; 3 Department of Molecular Oncology, Graduate School of Medicine, Tokyo Medical and Dental University, 1-5-45 Yushima, Bunkyo-ku, Tokyo, 113-8519 Japan

**Keywords:** Pancreatic cancer, DP-CAR, Arterial reconstruction, Chemoradiation therapy, Locally advanced, Gemcitabine, Nab-paclitaxel

## Abstract

**Background:**

Pancreatic cancer is a disease with a poor prognosis, requiring multidisciplinary treatment combining chemotherapy and surgery for effective management. Distal pancreatectomy with celiac axis resection (DP-CAR) is a surgical intervention performed for locally advanced pancreatic cancer, but the benefit of arterial reconstruction in DP-CAR is unclear.

**Case presentation:**

A 49-year-old man with pancreatic cancer was referred to our hospital. Imaging revealed a 54-mm tumor mainly in the pancreatic body, but with arterial infiltration including into the celiac, common hepatic, left gastric, splenic and gastroduodenal arteries. Distant metastases were not detected. The patient was diagnosed with unresectable locally advanced pancreatic cancer and chemoradiotherapy was planned. Three cycles of gemcitabine (1000 mg/m^2^) plus nab-paclitaxel (125 mg/m^2^) every 4 weeks were followed by irradiation (2 Gy/day, total 50 Gy over 25 days) together with S-1 administration (80 mg/m^2^/day). A partial response (PR) according to Response Evaluation Criteria in Solid Tumors (RECIST) was achieved, so surgical intervention was considered. Because the tumor had invaded the root of the gastroduodenal artery, we performed DP-CAR with resection of the gastroduodenal artery, followed by arterial reconstruction of the proper hepatic and left gastric arteries, anastomosed with the abdominal aorta using a great saphenous vein graft in the shape of a “Y”. Histopathology showed that 60% of tumor cells were destroyed by the chemoradiotherapy, defined as grade IIb in the Evans classification. No malignancy was detected at the surgical margin, including the celiac artery, gastroduodenal artery or pancreatic stump; thus R0 surgery was successful. S-1 (80 mg/day) was administered as adjuvant chemotherapy for 6 months. The patient is now doing well without recurrence for > 2 years after the initial treatment (more than 16 months after surgery).

**Conclusion:**

For locally advanced pancreatic cancer, multidisciplinary treatment combining gemcitabine/nab-paclitaxel-based chemoradiotherapy and then DP-CAR surgery with gastroduodenal artery resection and arterial reconstruction using saphenous vein grafting enabled R0 resection in this patient and led to a favorable long-term prognosis.

## Background

Pancreatic cancer (PC) is a fatal disease with high malignancy and a very poor prognosis. It is often advanced at the time of definitive diagnosis and surgical intervention is then challenging [1]. Effective chemotherapy for advanced PC, such as gemcitabine plus nab-paclitaxel (GEM/nab-PTX) or oxaliplatin, leucovorin, irinotecan, plus 5-fluorouracil (FOLFIRINOX) has been developed over the last decade [2, 3]. However, chemotherapy alone has not been sufficient to achieve long-term survival, and multidisciplinary treatment combining chemotherapy with surgical intervention improved prognosis for locally advanced unresectable (UR-LA) PC [4]. Moreover, some reports indicated that UR-LA PC could be radically resected after chemoradiotherapy and could result in prolonged survival [5, 6].

Distal pancreatectomy with celiac axis resection (DP-CAR) is a surgical method for locally advanced cancer of the pancreatic body. In this approach, the tissue surrounding the pancreas and retroperitoneum, including the celiac artery (CeA) and common hepatic artery (CHA), is removed to achieve R0 status [7]. Generally, gastroduodenal artery (GDA) preservation is required to maintain hepatic blood flow. Therefore, a condition for this surgery is that there must be no tumor invasion from the GDA to the proper hepatic artery (PHA).

Here, we present a successful case of DP-CAR with arterial reconstruction of the left gastric artery (LGA) and the PHA using great saphenous vein grafting (SVG) for UR-LA PC after chemoradiotherapy following chemotherapy with GEM/nab-PTX.

## Case presentation

A 49-year-old man complained of left abdominal pain. He had a past medical history of duodenal ulcer and appendicitis, no smoking history, and no alcoholic history. Abdominal ultrasonography revealed a 33-mm tumor located in the pancreatic body, and he was referred to our hospital for further evaluation and treatment.

**Fig. 1:**
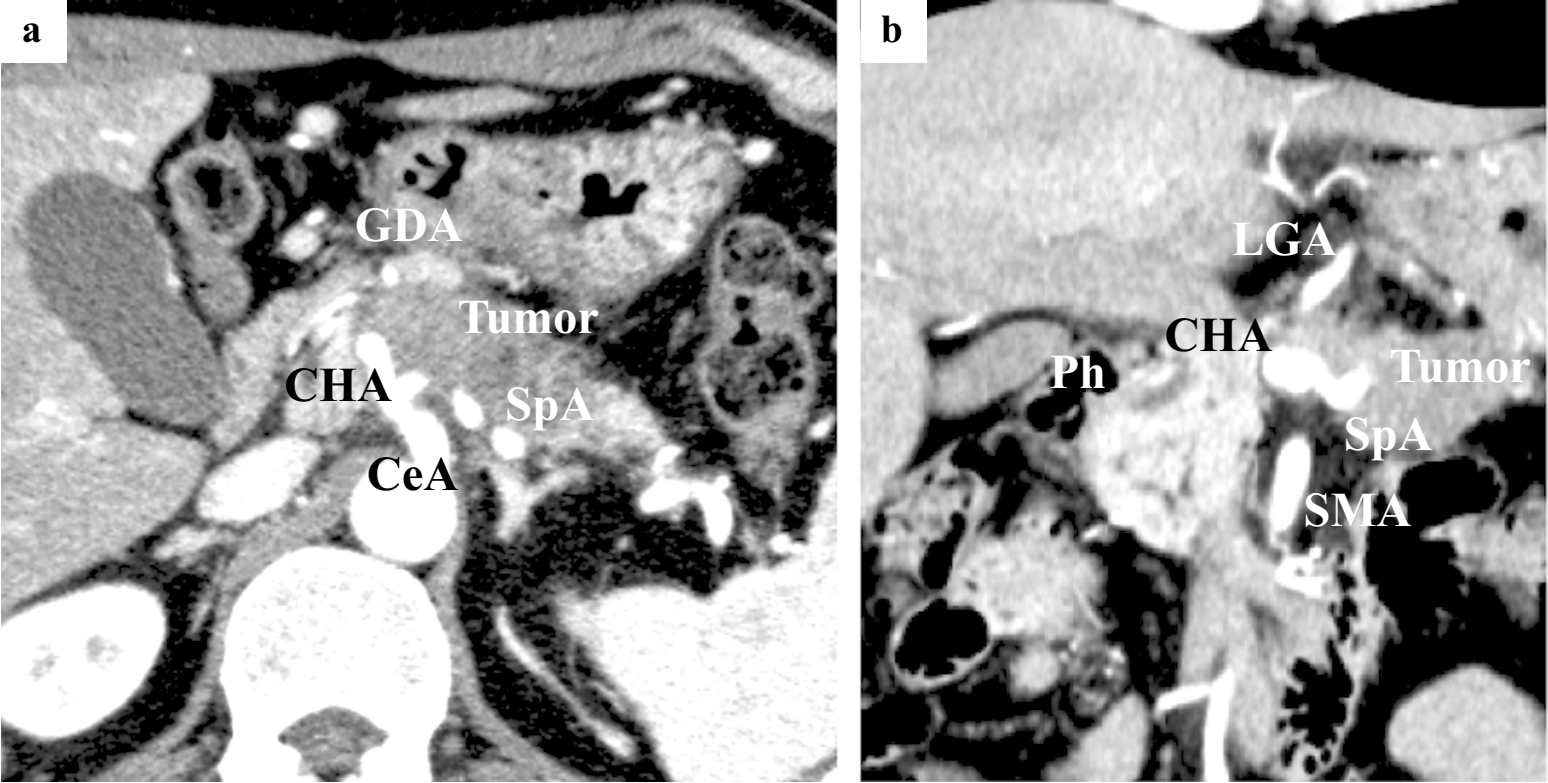
Abdominal CT imaging at the initial diagnosis. **a** 54-mm hypovascular tumor in the body to tail of the pancreas. **b** The main tumor in contact with the CeA, CHA, LGA, SpA and GDA

Laboratory data showed elevated tumor markers including CEA (6.0 ng/ml) and CA19-9 (72.9 U/ml). Computed tomography (CT) revealed a 54-mm hypovascular tumor in the body to tail of the pancreas (Fig. 1a). Direct tumor invasion was seen around the CeA, CHA, LGA, splenic artery (SpA), and GDA (Fig. 1b). There was no encasement around the superior mesenteric artery (SMA). No liver metastasis was found by gadoxetic acid-enhanced magnetic resonance imaging (EOB-MRI). Positron emission tomography (PET)–CT showed increased metabolic activity in the pancreas (maximum standardized uptake value for the tumor was 5.7), but there were no suspicions of metastases in other organs. Endoscopic ultrasonography-guided fine-needle aspiration from the pancreas body tumor was performed, and the pathological findings revealed pancreatic adenocarcinoma.

According to the National Comprehensive Cancer Network (NCCN) guidelines version 2.2018, resectability was determined as UR-LA. We planned conversion surgery (CS) after multidisciplinary treatment, including chemotherapy and chemoradiotherapy.

First, three courses of chemotherapy were proposed. The regimen was standard (days 1, 8, and 15: injection of GEM (1000 mg/m^2^) and nab-PTX (125 mg/m^2^) every 4 weeks). Adverse events were general fatigue and Grade 3 neutropenia, so chemotherapy was omitted twice (thus, 7 cycles were given). After chemotherapy, CT imaging showed tumor size reduction to 40 mm, indicating stable disease (SD) according to the Response Evaluation Criteria in Solid Tumors (RECIST). Next, we performed radiotherapy together with S-1 administration. Radiotherapy consisted of 2 Gy per fraction given 5 days per week for a total of 50 Gy. S-1 was administrated orally at 80 mg/m^2^/day for 28 consecutive days. This treatment was not accompanied by any significant adverse events.

**Fig. 2:**
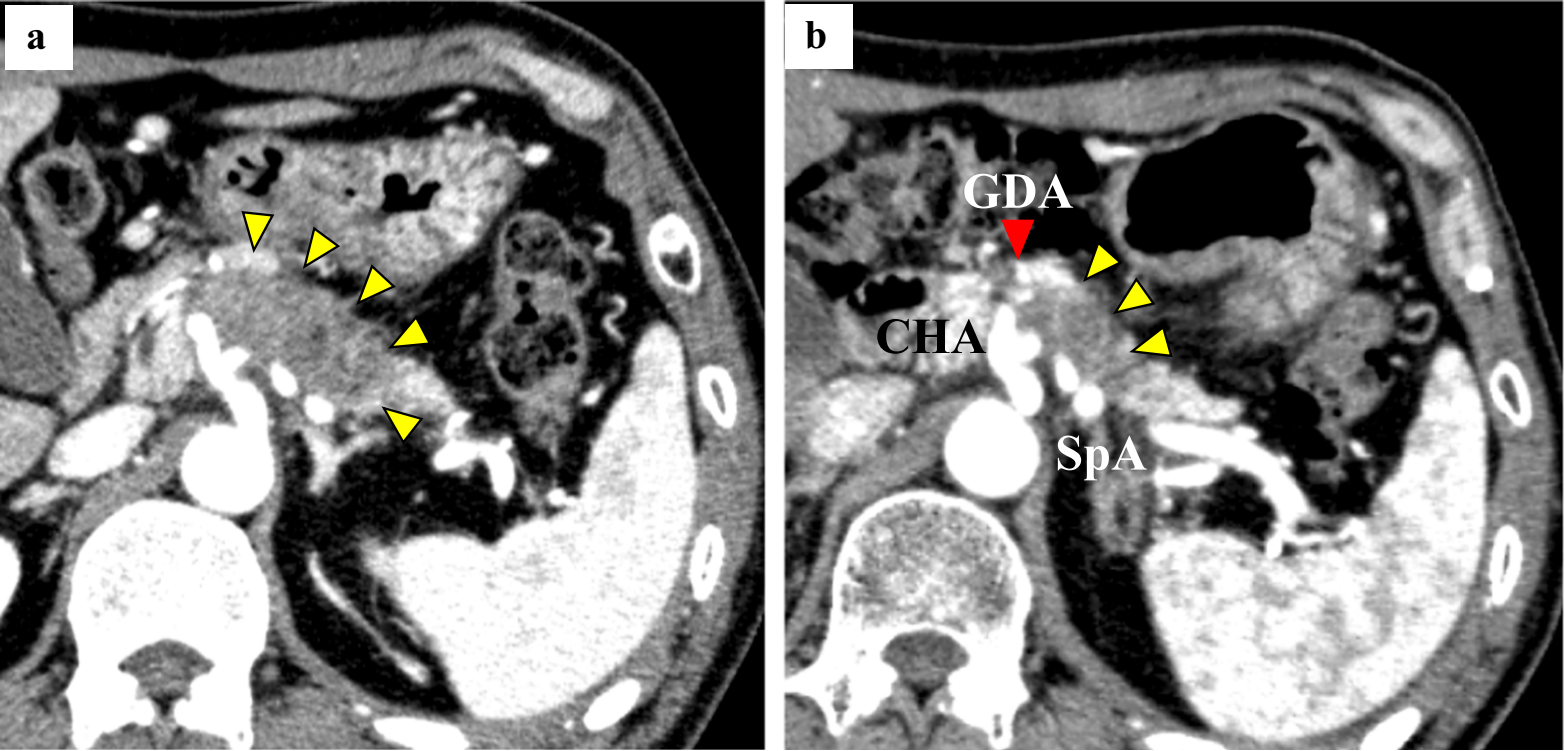
Abdominal CT changes before and after chemoradiotherapy. **a** Yellow arrows indicate the pancreatic tumor before chemoradiotherapy, and the red arrow indicates GDA. **b** After chemoradiotherapy, the size of the main tumor was reduced from 54 to 31 mm, but abnormal soft tissue shadows around main arteries were still detected

After chemoradiotherapy, tumor markers decreased to within the normal range (CEA: 2.6 ng/ml and CA19-9: 15.1 U/ml). CT imaging showed tumor size reduction to 31 mm, indicating partial response (PR) according to RECIST, but soft tissue density around CeA, CHA, LGA, SpA and GDA was still detected (Fig. 2). PET–CT imaging showed no suspicious distant metastases, and the maximum standardized uptake value for the pancreas body tumor was 2.2.

By angiography, we found that the left hepatic artery (LHA) branched off from the LGA, and the right hepatic artery (RHA) from the PHA. Proximal balloon occlusion of the CeA showed that RHA blood flow from the SMA was maintained. However, LHA blood flow could not be detected since the balloon blocked blood flow to the LGA, and a definite arterial connection between the right and left liver could not be observed.

In the preoperative evaluation, we considered that R0 status could be achieved by DP-CAR with arterial reconstruction, even if tumor invasion to the GDA or PHA had occurred. Surgery was then carried out 8 months after the initial start of treatment.

**Fig. 3:**
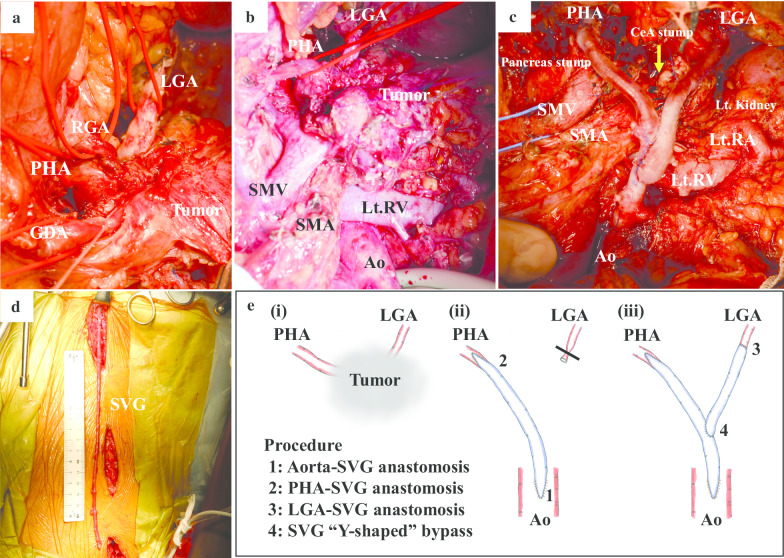
Intraoperative findings. **a** The tumor involved the surrounding arteries. **b** Plan to reconstruct arteries after dissecting the pancreas. **c** Image obtained after tumor resection and reconstruction of PHA and LGA. **d** Harvesting the SVG. **e** The details of the procedures in grafting. **e**-(i), (ii): after the aorta and the SVG graft was anastomosed, the PHA and the SVG graft was anastomosed. **e**-(iii): another SVG graft was anastomosed to the LGA. Finally, the first and second SVG were anastomosed together to form a Y-shape

Intraoperative findings revealed that the tumor was located mainly in the pancreas body, but had also invaded into the arteries around the pancreas, including the CeA, CHA, LGA, SpA, and GDA (Fig. 3a). The GDA, CHA, and LGA could not be preserved on surgery. Firstly, the GDA was resected proximal to the branch of the right gastroepiploic artery (RGEA). Pancreatic transection was performed using a scalpel, and the pancreatic stump was closed by suturing. The root of the right gastric artery (RGA) was also involved in the tumor, and the PHA was divided on the hepatic side from the RGA. This meant that the PHA was practically the same as RHA since the replaced LHA arose from the LGA. After RHA resection, the backflow from the RHA stump appeared weak with only a small amount of blood flow, though we expected RHA blood flow from intrahepatic communicating arcades. Ultrasonography after RHA resection showed little change in the right hepatic blood flow before and after the LGA was clamped, and the decreased communication between the left and right hepatic arteries was a concern. We decided that it was necessary to reconstruct both the LGA and RHA to ensure hepatic and gastric blood flow because gastric blood flow would be supplied by the RGEA alone, and intraarterial communication between the left and right hepatic arteries was a concern. Thus, we performed a bypass procedure between the abdominal aorta below the renal artery with the PHA using the right great saphenous vein grafting (SVG). After anastomosis between the aorta and PHA, a Y-shaped bypass was constructed and anastomosed with the LGA (Fig. 3b–e).

Intraoperative frozen section pathology findings confirmed tumor-free margins of the CeA, GDA and pancreas stump. Based on these findings, we performed DP-CAR with arterial reconstruction using SVG to achieve R0 resection. The total duration of surgery was 754 min, and blood loss was 660 ml.

**Fig. 4:**
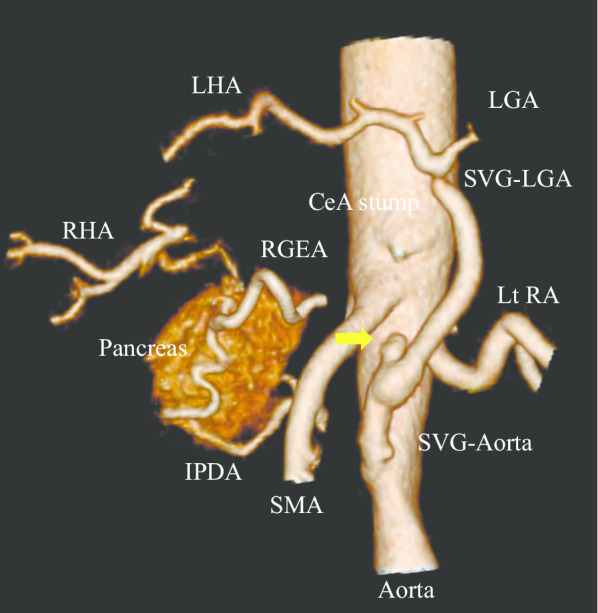
Vascular imaging of postoperative abdominal CT. Yellow arrow indicates the occlusion site of aorta–PHA grafting. Collateral blood flow from the pancreas to the liver was identified. *IPDA* inferior pancreaticoduodenal artery, *RA* renal artery, *RGEA* right gastroepiploic artery

Postoperative complications included decreased appetite, although no clinically significant pancreatic fistula was identified. Postoperative gastrointestinal endoscopy showed erythematous mucosal surface and stenosis from the pyloric region to the duodenal bulb. These complications were treated with drug therapy (Clavien–Dindo classification IIIa). Postoperative CT imaging clearly showed blood flow through the aorta–LGA bypass, but the aorta–PHA bypass did not (Fig. 4). The increase in liver enzymes was mild (max AST: 250 IU/l, ALT: 203 IU/l), and liver function was preserved. Thus, no antiplatelet medication or heparin was administrated. The patient was discharged 73 days after surgery.

**Fig. 5:**
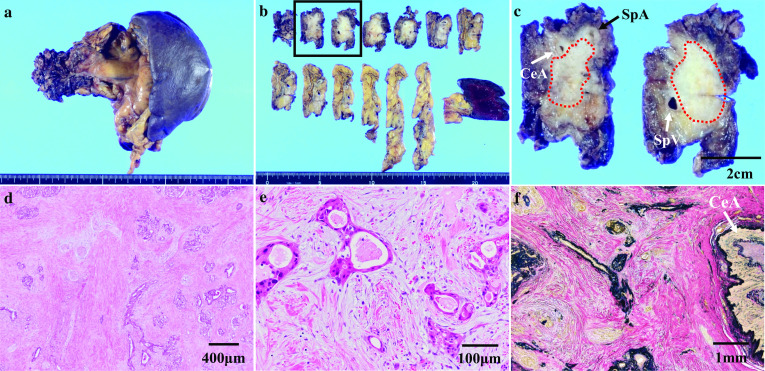
Histopathological findings. **a**, **b** 52-mm tumor in the body to tail of the pancreas. **c** Magnified view of the rectangle in **b**. Cancer cells were distributed within the fibrosis, surrounded by red dots. CeA and SpA were involved in the area. **d**, **e** In HE staining, about 60% of cancer cells were destroyed by chemoradiotherapy. **f** Immunohistochemistry (EVG staining) showed a severe fibrous adhesion around the celiac artery

Histopathological findings showed a 52-mm moderately differentiated tubular adenocarcinoma in the body to tail of the pancreas (Fig. 5a–c). Although cancer cells invaded the pancreatic nerve plexus, 60% of tumor cells were destroyed by chemoradiotherapy, defined as grade IIb in the Evans classification (Fig. 5d, e). Severe fibrosis most likely caused by degeneration of cancer cells after chemoradiation and fragmentation of the external elastic membrane around the CeA wall were observed, but there were no arterial cancer invasion (Fig. 5f). According to the Union for International Cancer Control (UICC) TNM classification (8th edition), the tumor was T3 N0 M0, Stage IIA. No residual cancer cells were detected at the surgical margin, including the CeA, GDA, and pancreas stump (i.e., R0 resection was achieved) (Fig. 4d, e).

The patient completed 6 months of S-1 adjuvant chemotherapy (80 mg/day) and has been doing well without recurrence for more than 2 years after the initial treatment (> 16 months after surgery).

## Discussion

PC is one of the deadliest cancers, and has a very poor prognosis [1]. Here, we report a patient for whom we performed DP-CAR with arterial reconstruction using SVG following multidisciplinary treatment, including GEM/nab-PTX chemotherapy and chemoradiotherapy. Relapse-free survival for more than 16 months after surgery was achieved. Hence, we believe that reporting the multidisciplinary treatment of this case is valuable.

Approximately 30–35% of patients with PC are initially diagnosed as UR-LA PC [1, 8]. It is difficult to treat such patients by surgery alone, and multidisciplinary treatment, including chemotherapy and radiotherapy, is required. Recently, several studies have reported initially UR-LA PC converting to resectable disease following tumor debulking by chemoradiotherapy [4, 5, 9–13]. GEM-based regimens including GEM + nab-PTX, FOLFIRINOX, or S-1-based regimens were frequently used as chemotherapy before such conversion surgery. Patients who received CS had a better prognosis relative to those who were not operable (median overall survival of CS vs non-CS: 22.1–39.7 vs 8–20.8 months). However, complications after CS were as high as 8.6–43% and relapse-free survival after CS was 12.3–22.5 months. Especially when DP-CAR was the selected CS, 3-year overall survival rates were reported as 65.5%, or the median overall survival time (MST) as 38.6 months, and morbidity was 27.3–42% [14, 15]. Thus, CS was considered to have a certain potential. In our institution, we apply the same criteria of CS for the treatment of UR-LA PC, as in this case. Firstly, we use GEM/nab-PTX as the basic regimen. After three courses of chemotherapy, radiation with S-1 administration is added for patients with controlled local disease. For patients without progressive disease according to RECIST on imaging after chemoradiotherapy, CS is performed in cases that are judged resectable based on performance status and changes in tumor markers.

Recent data suggested several factors influencing the beneficial prognosis after surgery. In a large cohort study, including PC with distant metastasis, preoperative CA19-9 level, lymph node involvement, metastasis status, and vascular involvement were all found to be prognostic factors for survival after CS [16]. Donahue et al. [17] reported that when patients had initially unresectable PC, surgery needed to be carefully considered on the basis of lack of disease progression, good functional status, and decreased CA19-9 (a sialylated Lewis antigen representing a quantitative biomarker used in PC). It was reported that survival after CS was prolonged for patients in whom CA19-9 had been significantly reduced or normalized following neoadjuvant therapy [18, 19]. Moreover, the duration of chemotherapy before CS was also an important factor related to prognosis. According to the report of Satoi et al., patients who underwent CS following non-surgical treatment lasting longer than 240 days had a good prognosis after surgery [12]. In our case, the duration of preoperative chemoradiotherapy was 8 months, during which the CA19-9 decreased to within the normal range, and according to RECIST tumor shrinkage indicated a PR. In the surgical specimen, no lymph node metastases containing viable cancer cells were present. Therefore, we speculated that prolonged survival could be achieved in our case and hence considered CS.

DP-CAR is a surgical procedure to ensure achieving negative margins of the celiac artery, periarterial plexus, and retroperitoneal tissue in locally advanced cancer of the pancreatic body [7]. Recent reports indicated that DP-CAR contributed to improving PC prognosis [20–23]. However, none of these previous reports had the LGA and PHA been reconstructed at the same time. We performed simultaneous reconstruction of the LGA and PHA as these arteries maintained the blood flow to the liver and stomach. One of the problems with arterial reconstruction in DP-CAR is the possibility of rupture and bleeding at the arterial anastomosis site following postoperative pancreatic fistula. For this reason, the indication for arterial reconstruction with DP-CAR must be carefully determined, and in some cases, total pancreatectomy should be considered to avoid postoperative hemorrhage due to pancreatic fistula [24]. In this case, the discussion took place preoperatively as well. As a result, we avoided total pancreatectomy to preserve pancreatic function considering the patient's age. In previous reports of arterial reconstruction with pancreatectomy, end-to-end arterial anastomosis was often chosen [25, 26]. However, we selected a bypass procedure between the abdominal aorta with PHA and LGA using the SVG formed into a Y-shape, because the SVG was of sufficient length to freely reconstruct complex arteries. Preoperatively, we had considered the middle colic artery as a candidate for reconstruction, but intraoperatively, we found that the mesentery of the transverse colon was hardened and thickened due to the effects of cancer invasion and radiotherapy, and the middle colic artery could not be used for reconstruction. CS for advanced PC often does not reveal a suitable artery for reconstruction. It is therefore important to have the option of arterial reconstruction using SVG when performing aggressive surgery for advanced PC.

On the other hand, reconstruction using the SVG carries problems of graft patency or varicose veins due to long-term arterial pressure. No coherent reports have showed the incidence of graft occlusion or varicose veins in the SVG after abdominal arterial reconstruction. However, the incidence of varicose veins was reported to be less than 1% in coronary artery bypass grafting using the SVG [27], and the frequency of varicosity of SVG after femoropopliteal artery bypass surgery was 3.7% [28]. In our case, postoperative CT imaging revealed no SVG varicose veins, and the LGA was patent, but this could not be confirmed for the PHA. As shown in Fig. 4, blood flow of the LHA and RHA were confirmed. Possible reasons for PHA graft occlusion were the collateral circulation providing sufficient hepatic blood flow, and minor pancreatic fistula, although clinically insignificant, affecting graft patency. Although liver function was relatively well-preserved, suspected symptoms of ischemic gastropathy (IG) including decreased appetite were seen. Current studies reported that LGA resection is a significant risk factor for IG [29, 30], and that DP-CAR with preservation or reconstruction of the LGA might reduce its risk [31]. In our case, the GDA and LGA were resected to achieve R0 status, and the PHA and LGA were reconstructed. Postoperative CT showed that the LGA and RGEA were maintained, suggesting sufficient arterial blood flow to the stomach. Thus, changes in venous blood flow were considered as a probable cause of such symptoms. Resection of the main veins around the stomach may have caused congestion and edema of the gastric mucosa, resulting in pyloric stenosis. Without reconstruction of the LGA, more severe complications such as gastric mucosal necrosis might have occurred because of inadequate arterial blood flow. Therefore, we presume that it is necessary to reconstruct the LGA when the GDA is dissected in DP-CAR, and that reconstruction of the arteries contributes to the reduction of complications.

## Conclusions

For UR-LA PC after chemoradiotherapy, DP-CAR was achieved after GDA resection in this case by reconstruction of the PHA and LGA using the SVG. This treatment strategy could be an excellent option for very advanced PC such as UR-LA PC with arterial reconstruction after pancreatectomy.

## Data Availability

All data generated during this report are included in this published article.

## Abbreviations

CeA: Celiac artery
CHA: Common hepatic artery
CT: Computed tomography
DP-CAR: Distal pancreatectomy with celiac axis resection
EOB-MRI: Gadoxetic acid-enhanced magnetic resonance imaging
FOLFIRINOX: Oxaliplatin, leucovorin, irinotecan, plus 5-fluorouracil
GDA: Gastroduodenal artery
GEM: Gemcitabine
LGA: Left gastric artery
LHA: Left hepatic artery
nab-PTX: Nab-paclitaxel
NCCN: National Comprehensive Cancer Network
PC: Pancreatic cancer
PET: Positron emission tomography
PHA: Proper hepatic artery
RECIST: Response Evaluation Criteria in Solid Tumors
RGA: Right gastric artery
RGEA: Right gastroepiploic artery
RHA: Right hepatic artery
SMA: Superior mesenteric artery
SpA: Splenic artery
SVG: Saphenous vein graft
UICC: Union for International Cancer Control
UR-LA: Locally advanced unresectable
